# Cardiovascular Outcomes of Disease-Modifying Antirheumatic Drugs in Rheumatoid Arthritis: A Review of the Current Evidence

**DOI:** 10.7759/cureus.82269

**Published:** 2025-04-14

**Authors:** Priya Sunkara, Naga Alekhya Garikipati, Rithish Nimmagadda, Anjani Mahesh Kumar Cherukuri, Himaja Anne, Rumitha Chakilam, Deepesh Yadav

**Affiliations:** 1 Internal Medicine, White River Health, Arkansas, USA; 2 Medicine, MediCiti Institute of Medical Sciences, Hyderabad, IND; 3 Internal Medicine, One Brooklyn Health, New York, USA; 4 Internal Medicine, Rithish Nimmagadda, Hyderabad, IND; 5 Medicine, Guntur Medical College, Guntur, IND; 6 Medicine, Hind Institute of Medical Sciences, Sitapur, IND; 7 Internal Medicine, Gandhi Hospital, Secunderabad, IND; 8 Rheumatology, University of Arkansas for Medical Sciences, Little Rock, USA; 9 Orthopedic Surgery, Kathmandu University, Dhulikhel, NPL; 10 Internal Medicine, Hurley Medical Center, Michigan State University College of Human Medicine, Flint, USA

**Keywords:** cardioprotective effects, cardiovascular disease, rheumatoid arthritis, tnf inhibitors, traditional dmards

## Abstract

Rheumatoid arthritis (RA) significantly increases the risk of cardiovascular disease (CVD), including myocardial infarction (MI), stroke, and heart failure (HF), with RA treatments influencing cardiovascular outcomes. This review analyses the cardiovascular effects of methotrexate, leflunomide, hydroxychloroquine, sulfasalazine, and TNF inhibitors (infliximab, etanercept, adalimumab, and certolizumab) in RA management, emphasizing their safety and risks in CVD. This narrative literature review was conducted using searches of the PubMed database from inception through January 2025. We included meta-analyses, systematic reviews, randomized controlled trials, observational studies, pharmacovigilance studies, and animal studies. Methotrexate offers cardiovascular benefits by reducing inflammation and improving endothelial function. However, it also raises homocysteine levels, which promote oxidative stress and endothelial injury - effects that can be mitigated by folic acid supplementation. Leflunomide’s cardiovascular effects remain poorly defined, highlighting the need for further research. Hydroxychloroquine may prolong the QT interval, raising the risk of conduction disorders and necessitating monitoring in high-risk patients. Sulfasalazine shows potential cardiovascular benefits by inhibition of platelet aggregation, improved endothelial function, and reduced lipid levels, although more research is needed for conclusive evidence. TNF inhibitors, such as infliximab, etanercept, adalimumab, and certolizumab pegol, reduce inflammation-driven cardiovascular risks but are contraindicated in patients with severe HF (New York Heart Association [NYHA] classes III and IV).

## Introduction and background

Rheumatoid arthritis (RA) is a chronic, systemic, autoimmune inflammatory disease that primarily affects the joints and periarticular soft tissues. It is characterized by symmetrical polyarthritis, particularly involving the small joints of the hands and feet, leading to pain, swelling, stiffness, and progressive joint destruction and deformity [1]. RA is the most common form of inflammatory arthropathy and a leading cause of disability worldwide, resulting in joint deformities, stiffness, pain, and loss of mobility [2]. Over the past 30 years, the burden of RA has risen, and by 2040, global RA cases are projected to rise 1.4-fold, from 1.07 million in 2019 to approximately 1.5 million [3].

The pathogenesis of RA involves a multifaceted interplay of genetic, environmental, and immunologic factors, leading to chronic inflammation, autoantibody production, and joint destruction. The process begins when dendritic cells, macrophages, and B cells present antigens, including citrullinated proteins to T cells. This activates T cells to produce cytokines, which then trigger further immune responses by activating monocytes, macrophages, and synovial fibroblasts, releasing proinflammatory cytokines. 

Key cytokines in RA, such as tumor necrosis factor (TNF)-α and interleukin (IL)-6, play central roles in inflammation. Cardiovascular disease (CVD) is the leading cause of death in patients with RA, accounting for approximately 40% of deaths due to increased risk driven by systemic inflammation [4]. 

The development of CVD in patients with RA is driven by a combination of traditional and non-traditional risk factors, with chronic systemic inflammation being central. Elevated levels of proinflammatory cytokines like TNF-α, IL-6, and IL-1 contribute to endothelial dysfunction, oxidative stress, and the development of atherosclerosis [5]. Endothelial dysfunction, a key early event in atherosclerosis, is characterized by reduced vasodilation, increased adhesion molecule expression, and a prothrombotic state, all of which promote the formation and instability of atherosclerotic plaques [6]. Additionally, RA-induced systemic inflammation leads to disruptions in lipoprotein metabolism, known as the *lipid paradox*, where patients have lower levels of total cholesterol and high-density lipoprotein (HDL), but still face a higher risk of CVD due to dysfunctional HDL and increased oxidation of low-density lipoprotein (LDL) [7]. The persistent inflammatory state further impairs the regenerative capacity of endothelial progenitor cells, worsening endothelial injury and the progression of atherosclerosis [8].

In conclusion, CVD in patients with RA arises from a complex interplay of chronic inflammation, endothelial dysfunction, altered lipoprotein metabolism, and increased myelopoiesis, all of which accelerate atherosclerosis and elevate cardiovascular risk. Our growing understanding of the pathogenesis of RA has provided valuable insights into the mechanisms behind existing therapies and paved the way for new treatments that more effectively manage disease activity and associated comorbidities, including cardiovascular events.

This study will explore the comparative effectiveness, safety profiles, and impact on cardiovascular health of traditional and biologic disease-modifying antirheumatic drugs (DMARDs), offering insights into how these therapies address both the inflammatory components of RA and its associated comorbidities, including the elevated risk of cardiovascular events.

## Review

DMARDs, used to modify the disease course and prevent joint damage in RA, are categorized into traditional (e.g., methotrexate [MTX], sulfasalazine, hydroxychloroquine, and leflunomide) and biologic DMARDs (e.g., TNF-α inhibitors [TNFis], IL-6 inhibitors, and B-cell depletion agents) [9]. This analysis compares the cardiovascular safety profiles of conventional DMARDs and anti-TNF biologics, underscoring the need to understand these differences for optimal RA treatment.

Methotrexate

MTX has been widely used since the late 1980s for treating RA. It is recommended as the first-line treatment by the American College of Rheumatology and the European League Against Rheumatism for early and established RA [10]. MTX is a structural analogue of folic acid. It inhibits dihydrofolate reductase, preventing the formation of tetrahydrofolate, which is necessary for nucleotide and DNA synthesis, thereby interfering with cell division and proliferation. This suppresses T-cell proliferation and other immune responses while promoting adenosine release [10].

The cardioprotective effect of MTX in RA arises primarily from its anti-inflammatory properties rather than direct action on atherosclerotic lesions. MTX inhibits pro-atherosclerotic cytokines like TNF-α, IL-1, and IL-6, reducing systemic inflammation, which helps mitigate endothelial dysfunction and atherosclerosis progression. Additionally, it facilitates cholesterol efflux from macrophages through adenosine release, reducing foam cell formation and lowering atherosclerosis risk [11]. Several cohort studies support MTX’s cardioprotective effects. For instance, a cohort study in the United States, involving 1,240 patients observed for six consecutive years, demonstrated about 70% reduction in the cardiovascular mortality and 60% overall mortality risk (*P*-value = 0.02) [12,13]. Another cohort study conducted in the United States in 2008 with 16,752 patients with RA who were treated with MTX showed a decreased risk of CVD with a risk reduction of 35% (95% confidence interval [CI] 0.59-0.72) [14].

In 2006, a case-control study with 613 patients with RA (72 with CVD, 541 without) found that MTX monotherapy or combination therapy effectively suppressed inflammation, reducing atherosclerosis and CVD risk [15]. In 2006, the Questionnaires in Standard Monitoring of Patients with Rheumatoid Arthritis Program (QUEST-RA) project, a multinational cross-sectional study conducted in the United States with 4,363 patients from 48 sites in 15 countries, found that prolonged MTX usage was associated with decreased prevalence of stroke and myocardial infarction (MI) [16].

In 2011, a large U.S. registry study involving 10,156 patients found no reduction in CVD risk with methotrexate compared to other DMARDs; however, several limitations may affect the reliability of these findings [17]. As an observational study, it may suffer from selection bias due to reliance on the MarketScan database. The short follow-up period and lack of randomization limit the ability to establish causality, while potential misclassification of DMARD exposure and CVD outcomes further bias the results. These limitations suggest that the findings should be interpreted cautiously, emphasizing the need for more robust evidence from randomized controlled trials (RCTs) or well-conducted longitudinal studies.

In 2021, a meta-analysis by Sun et al., including a total of 195,416 patients with RA, found that MTX use significantly reduces cardiovascular events (MI and stroke), with an RR of 0.798 (95% CI 0.726-0.876, *P* = 0.001, *I*² = 27.9%) [18]. Similarly, in 2011, Mitch et al.'s meta-analysis, including 66,334 individuals with 6,325 CVD events, showed a 21% reduction in CVD risk with MTX, with an RR of 0.76 (95% CI: 0.69-0.84) for CVD and 0.70 (95% CI 0.56-0.87) for stroke, with minimal heterogeneity [19]. MTX use in patients with RA is associated with significantly lower carotid intima-media thickness (CIMT) through activation of the AMP-activated protein kinase (AMPK) pathway, suggesting a reduced risk of atherosclerosis [20]. A 2015 cross-sectional study by Kisiel et al. found that higher doses (>20 mg/week) further amplified this effect, reducing CIMT and plaque prevalence. This dosage was selected based on clinical guidelines and evidence supporting its effectiveness in managing RA [21].

MTX shows a positive cardioprotective effect on patients with RA, but folic acid levels should be monitored and supplemented to prevent the increased homocysteine concentration in the body. The cardioprotective effects of MTX are mediated through several mechanisms, including anti-inflammatory properties, improvement of endothelial function, activation of AMPK, antioxidant effects, and enhancement of HDL cholesterol efflux capacity, along with a reduction in foam cell formation. However, many aspects of its mechanisms remain poorly understood. Further research through RCTs is needed to establish the extent of MTX's impact on cardiovascular risk in patients with RA.

Leflunomide

Leflunomide is a non-biological DMARD belonging to the isoxazole derivative class, and it exerts effects on inflammation and immunosuppression. Its mechanism of action involves the inhibition of dihydroorotate dehydrogenase, which is crucial for de novo pyrimidine synthesis in rapidly dividing cells, particularly activated T-cells. This inhibition reduces T-cell proliferation, leading to a subsequent decrease in inflammation and joint damage. Leflunomide is the immunomodulatory medication that metabolizes in the body to form an active metabolite A77-1726, also called teriflunomide [10]. Various studies have shown that A77-1726 can alleviate myocardial hypertrophy induced by pressure overload or angiotensin and also prevent cardiac fibrosis by inhibiting the conversion of cardiac fibroblasts into muscle fibroblasts. Leflunomide, known for its potential to increase blood pressure, was shown in a two-year RCT involving 482 patients to elevate both systolic and diastolic levels. These findings are consistent with its U.S. Food and Drug Administration (FDA)-approved side effect profile, compared to MTX and placebo [22].

In 2024, an experimental animal study by Jiang et al. explored the mechanisms by which leflunomide may attenuate atherosclerosis and improve cardiovascular outcomes by regulating lipid metabolism and endothelial dysfunction via the dihydroorotate dehydrogenase (DHODH)/AMPK signaling pathway [23]. Despite these promising findings, limited studies on leflunomide's impact on cardiovascular events mean that further large-scale studies are needed to fully understand its cardioprotective effects.

Hydroxychloroquine

Hydroxychloroquine (HCQ) is a non-biological DMARD that disrupts autophagy and lysosomal activity. Lysosomes play a key role in cellular recovery, including in immune cells, and reducing their activity leads to anti-inflammatory and immune-modulatory effects. HCQ also inhibits the production of IL-1, IL-6, TNF-α, and IFN-γ, which contributes to a reduction in inflammation [10]. A retrospective cohort study conducted in the United States from 2001 to 2013, with 1,266 patients with RA (547 HCQ users and 719 non-users) showed that HCQ use reduces the CVD risk by 72%, with a hazard ratio of 0.28 (95% CI 0.12-0.63, *P *= 0.002) [24]. HCQ also inhibits erythrocyte and platelet aggregation, prevents thrombosis, improves insulin sensitivity, and reduces total blood cholesterol, thereby reducing the CVD risk [24].

In 2018, a systematic review and meta-analysis of 16 studies demonstrated that HCQ may improve the metabolic profile, including lipid levels and diabetes incidence, which in turn could reduce the risk of cardiovascular events in patients with RA [25].

However, experimental research demonstrated that prolonged use of HCQ can cause serious heart diseases by blocking sodium and calcium channels, affecting membrane stability, prolonging the QT interval, widening the QRS complex, and causing conduction disorders [26]. So, regular EKG monitoring is recommended for patients with a history of heart failure (HF) on HCQ to mitigate the risk of cardiovascular events. This approach aligns with the guidelines from the American College of Rheumatology [10].

Sulfasalazine

Sulfasalazine exerts its anti-inflammatory and immunomodulatory effects in RA through its metabolism into sulfapyridine and 5-aminosalicylic acid (5-ASA). It inhibits the release of pro-inflammatory cytokines, such as IL-1, IL-6, and TNF-α, and suppresses the activation of nuclear factor kappa B (NF-κB), a key regulator of inflammation. Additionally, sulfasalazine inhibits secretory phospholipase A2 (sPLA2) and promotes adenosine release, further contributing to its anti-inflammatory actions [27]. In terms of cardiovascular safety, a preclinical ex vivo animal study has shown that sulfasalazine may have cardioprotective effects by inhibiting platelet aggregation and activating the nuclear factor erythroid 2-related factor 2 (Nrf2) signaling pathway. This reduces oxidative stress, improves endothelial function, and lowers blood lipid levels [28]. Sulfasalazine is frequently used in combination with other DMARDs, such as MTX and hydroxychloroquine, in patients with RA. 

In 2006, a case-control study of 613 RA patients (72 with CVD, 541 without) demonstrated that sulfasalazine, especially in combination with MTX, was associated with a significantly lower risk of CVD compared to patients who never used these DMARDs [15].

In 2002, an RCT with 171 patients concluded that triple combination therapy of MTX, sulfasalazine, and hydroxychloroquine was well tolerated and more effective than double combination therapies of MTX with sulfasalazine, MTX with hydroxychloroquine, or MTX alone [29].

However, there are no studies on sulfasalazine alone that can conclusively establish its protective effect on cardiovascular health, and more long-term studies are needed.

TNF-α inhibitors

TNFis are biological DMARDs used in RA to block TNF-α, reducing inflammation, modulating immune responses, lowering pro-inflammatory cytokines, and preventing cartilage damage. They are used as second-line therapy after traditional DMARDs fail. TNFi also offers cardiovascular benefits by reducing systemic inflammation. By suppressing TNF-α, they lower inflammatory markers like CRP and IL-6, which are linked to cardiovascular risk. This helps slow atherosclerosis, stabilize plaques, and reduce arterial stiffness, providing protection against cardiovascular complications in autoimmune diseases [30]. 

In patients with RA, TNFi therapy has been shown to improve endothelial function by enhancing nitric oxide (NO) availability, aiding vasodilation, and reducing vascular resistance. This, in turn, reduces aortic inflammation and stiffness, which are important predictors of cardiovascular events [31,32]. TNFi treatment has been shown to lower the risk of a first cardiovascular event by around 50% in patients with RA, based on a pooled analysis of several studies [33]. 

TNFis target inflammatory pathways in RA but have complex effects on cardiac myocytes [34]. TNF-α interacts with two receptors: TNFR1, which promotes apoptosis and negative inotropic effects, and TNFR2, which supports cell survival and cardioprotection. Blocking TNF-α indiscriminately may inhibit TNFR2’s beneficial effects, potentially worsening HF [35].

Infliximab

Infliximab is a chimeric monoclonal antibody that binds both soluble and membrane-bound TNF-α, neutralizing its pro-inflammatory effects. An RCT shows that infliximab improves arterial stiffness, as measured by pulse wave velocity, in patients with RA over 56 weeks. This improvement in vascular function could potentially translate to cardiovascular benefits. However, concerns over cardiovascular safety, particularly in patients with HF, remain [36].

In 2003, the Anti-TNF-α Therapy Against Congestive Heart Failure (ATTACH) trial enrolled 150 patients with HF (NYHA class III, EF < 35%), who were randomized to receive either a placebo, infliximab 5 mg/kg, or infliximab 10 mg/kg via IV infusion at weeks 0, 2, and 6. The primary endpoint was clinical status at 14 and 28 weeks. While both infliximab groups showed suppression of inflammatory markers and an initial increase in LVEF at week 14, the benefit was not sustained by week 28. An increase in worsening clinical status, deaths, and hospitalizations was observed, particularly in the higher-dose infliximab group. By week 28, a dose-dependent increase in adverse outcomes (worsening clinical status, hospitalizations, and deaths) was particularly evident in the high-dose group (10 mg/kg). These findings suggest a potential dose-related cardiotoxicity in patients with HF and have led to caution against infliximab use in this population [37]. It is important to note that the ATTACH trial was conducted in patients with advanced HF, who differ significantly from the general RA population, particularly those without overt CVD.

A real-world, observational cohort and registry-based study conducted in 2017 in the United Kingdom, involving 14,258 treated patients, reported that TNFi may reduce overall cardiovascular risk in RA patients without heart failure by lowering systemic inflammation - a key contributor to atherosclerosis and myocardial dysfunction [38]. Nonetheless, its use in patients with pre-existing heart conditions should be approached with caution.

Etanercept

Etanercept is a fusion protein that binds to soluble TNF-α, preventing receptor interaction. Unlike other TNFis, it does not directly target membrane-bound TNF-α, which may contribute to differences in safety profiles compared to other TNFis. 

A clinical trial conducted in 2009 in the United Kingdom with 148 RA patients showed that etanercept reduces arterial stiffness and pulse wave deflection, suggesting a potential reduction in cardiovascular morbidity [39]. Another clinical trial conducted in 2012, involving 48 patients (etanercept group: *n* = 28), found that etanercept significantly decreased the left ventricular mass index over six months, indicating a potential reduction in cardiovascular risk [40].

In 2002, the RENEWAL (Randomized Etanercept Worldwide Evaluation) study combined results from the RENAISSANCE (Randomized Etanercept North American Strategy to Study Antagonism of Cytokines) trial in North America and the RECOVER (Research into Etanercept Cytokine Antagonism in Ventricular Dysfunction) trial conducted in Europe, Israel, and Australasia. The study, involving 25 countries, evaluated etanercept in patients with heart failure (NYHA classes II-IV) and LVEF ≤30%. Patients were assigned to placebo or etanercept (25 mg once, twice, or thrice a week). The primary endpoint was clinical status at 24 weeks, with no significant differences observed between the placebo and etanercept groups in either study. Hazard ratios indicated slightly better outcomes with higher doses in the RECOVER trial but worse outcomes in the RENAISSANCE trial. Both studies were terminated in March 2001 due to a lack of benefit on morbidity and mortality. The studies suggested exercising caution when using etanercept in heart failure, particularly in patients under 65 or those with non-ischemic heart disease [41]. This study was conducted on patients with HF, limiting its applicability to the broader RA population.

While it does not appear to increase cardiovascular risk, caution should still be exercised in patients with pre-existing cardiac dysfunction [36]. Additional long-term studies are needed to further clarify its cardiovascular safety profile.

Adalimumab

Adalimumab is a fully human monoclonal antibody that specifically targets TNF-α, reducing inflammation in patients with RA. It is one of the most widely used TNFis due to its efficacy and generally favorable safety profile.

In 2024, a retrospective pharmacovigilance study utilized the FDA Adverse Event Reporting System (FAERS) database to evaluate cardiovascular adverse events (AEs) linked to TNFis. Of all TNFis, adalimumab is the only TNFi that showed an increased risk of thrombotic (arterial thrombus) cardiovascular events. The study adjusted for confounding factors by utilizing the adjusted reporting odds ratio (ROR) for potential confounds such as other medications and disease severity, providing a more accurate measure of the association between TNFis and cardiovascular AEs. It is important to note that this study is retrospective and observational and may have reporting bias. Additionally, the study used a disproportionality analysis to evaluate cardiovascular events. However, further RCTs are needed to draw definitive conclusions [42].

However, while adalimumab does not appear to exacerbate HF, its use in patients with severe cardiac dysfunction should still be monitored. Since TNF-α plays a complex role in cardiac remodeling, more studies are needed to determine whether long-term TNF blockade has any subclinical effects on myocardial function.

Certolizumab pegol

Certolizumab pegol, a PEGylated TNFi lacking an Fc region, may have a distinct safety profile, particularly regarding cardiovascular risk [43].

A retrospective study analyzing data from a claims database, which included 113,677 patients, found that anti-TNF therapy in patients with RA significantly reduced the risk of cardiovascular events. Each additional 6 months of therapy reduced the risk by 12%, with larger reductions (21%, 38%, and 51%) observed after 1, 2, or 3 years of use. The benefit was most notable in patients aged ≥50 and those without prior MTX use [44]. However, while TNFi may have a favorable cardiovascular profile, its use in patients with congestive heart failure (CHF) should be approached with caution due to limited studies and the general contraindications of TNFis in moderate-to-severe HF. Further research is needed to assess its impact on cardiovascular outcomes compared to other TNF inhibitors. 

Golimumab

Golimumab, a fully human monoclonal antibody that targets TNF-α, has shown strong efficacy in RA, but its cardiovascular effects are still being studied. A 2013 clinical trial, comprising two phase 3 studies (GO-BEFORE and GO-FORWARD), assessed the impact of golimumab, with or without MTX, on serum lipids and cardiovascular inflammatory markers in patients with RA. The results indicated that golimumab + MTX increased total cholesterol (TC), high-density lipoprotein (HDL), and low-density lipoprotein (LDL) compared to MTX alone at week 14 (GO-FORWARD) and week 24 (GO-BEFORE) [45]. Despite these elevations, atherogenic indices, such as the TC:HDL and LDL:HDL ratios, remained stable, suggesting a neutral cardiovascular risk profile. Notably, golimumab also improved LDL particle composition and significantly reduced inflammatory markers associated with CVD, with these effects sustained through week 52. These findings suggest a potential anti-inflammatory benefit without increasing lipid-related cardiovascular risk.

Despite these positive effects on inflammation and lipid profiles, all TNFis carry risks, particularly in patients with HF. Therefore, while golimumab has favorable effects on inflammation and lipid profiles in patients with RA, its use requires careful consideration of potential cardiovascular risk [46]. 

A concise summary of all the RA treatments discussed above is presented in Table 1.

**Table 1:** Cardiovascular impact of DMARDs: a mechanistic overview. DMARD, disease-modifying antirheumatic drug; CV, cardiovascular; TNF-α, tumor necrosis factor-alpha; IL-1, interleukin-1; IL-6, interleukin-6; EKG, electrocardiogram; 5-ASA, 5-aminosalicylic acid; HCQ, hydroxychloroquine; CVD, cardiovascular disease; RA, rheumatoid arthritis; HF, heart failure

DMARD type	Name of the drug	Mechanism of action	Effect on CV events	CV risk
Traditional DMARDs	Methotrexate	By inhibiting dihydrofolate reductase, interfering with nucleotide synthesis and T-cell proliferation, promotes adenosine release. [10]	Anti-inflammatory properties, reduces proatherosclerotic cytokines (TNF-α, IL-1, IL-6), improves cholesterol efflux, and decreases the risk of atherosclerosis.	Cardioprotective, but folic acid should be monitored and supplemented.
	Leflunomide	By inhibiting dihydro-orate dehydrogenase and reducing T-cell proliferation	Alleviates hypertrophy of the myocardium, prevents cardiac fibrosis, and increases hypertension [10]	Potentially cardioprotective but should monitor hypertension. Further large-scale studies are needed [10]
	Hydroxychloroquine	Inhibits autophagy, lysosomal activity, and production of inflammatory cytokines	Inhibits erythrocyte and platelet aggregation, prevents thrombosis, and reduces total cholesterol [24]	Cardioprotective; monitor EKG for QT prolongation [26].
	Sulfasalazine	Metabolized into sulfapyridine and 5-ASA, suppressing pro-inflammatory cytokines	Inhibition of platelet aggregation, improved endothelial function, and reduced lipid levels [28]	Cardioprotective when used with methotrexate and HCQ [29]. Limited standalone studies on CVD risk
Biologic DMARDs	Infliximab	Chimeric monoclonal antibody binding soluble and membrane-bound TNF-α	Improve arterial stiffness and vascular function in RA patients [36]	Cardioprotective; decreased overall CV risk in patients with RA without HF. Lower doses showed improvement in left ventricular function [37].
	Etanercept	Soluble TNF receptor inhibitor with less direct effect on TNFR1-mediated apoptosis	Reduce arterial stiffness, left ventricular mass index, and overall CV morbidity in RA [40]	Cardioprotective in patients without HF [41]
	Adalimumab	Fully human monoclonal antibody targeting TNF-α.	May decrease the atherosclerotic burden	Inconclusive; no significant increase in CV risk. One study showed increased thrombotic events [42], but no prospective RCT has confirmed a CV risk increase to date.
	Certolizumab pegol	PEGylated TNF inhibitor, lacking an Fc region, reduces antibody-dependent cytotoxicity.	Favorable CV profile due to reduced systemic inflammation [44]	Cardioprotective; may reduce systemic inflammation and improve CV outcomes in patients with RA [44]
	Golimumab	Fully human monoclonal antibody targeting TNF-α	Improving inflammatory markers and lipid profiles	Cardioprotective; GO-BEFORE and GO-FORWARD trials found improved CV markers, but caution is needed in patients with HF [45]

## Conclusions

This analysis compares the cardiovascular safety profiles of conventional synthetic DMARDs (csDMARDs) and TNFis in RA treatment. MTX remains the most supported csDMARD for cardiovascular benefit, although monitoring folic acid levels is necessary to prevent elevated homocysteine. Other agents like leflunomide, sulfasalazine, and hydroxychloroquine show some cardiovascular benefits, but the evidence is less robust due to fewer RCTs and smaller sample sizes. TNF inhibitors, including infliximab, etanercept, adalimumab, certolizumab pegol, and golimumab, effectively reduce systemic inflammation, a key driver of atherosclerosis and CVD, and improve outcomes such as reduced risks of myocardial infarction and cerebrovascular events. However, TNFis pose significant risks in patients with pre-existing HF, and the 2021 ACR guidelines recommend switching to non-TNF biologics or targeted synthetic DMARDs in patients with RA and HF (NYHA classes II-IV or EF < 50%). These findings highlight the importance of personalized treatment, incorporating cardiovascular risk stratification, QT interval assessment, existing cardiac conditions, and prior CVD history to optimize RA management while ensuring cardiovascular safety.
